# Estimating the disutility of relapse in relapsing–remitting and secondary progressive multiple sclerosis using the EQ-5D-5L, AQoL-8D, EQ-5D-5L-psychosocial, and SF-6D: implications for health economic evaluation models

**DOI:** 10.1007/s11136-023-03486-y

**Published:** 2023-07-31

**Authors:** Hasnat Ahmad, Julie A. Campbell, Ingrid van der Mei, Bruce V. Taylor, Qing Xia, Ting Zhao, Andrew J. Palmer

**Affiliations:** 1 Menzies Institute for Medical Research, University of Tasmania, Hobart, Australia; 2 Australian Government Department of Health and Aged Care, Canberra, Australia; 3 Australian Centre for Health Services Innovation and Centre for Healthcare Transformation, School of Public Health & Social Work, Queensland University of Technology, Brisbane, QLD Australia

**Keywords:** Multiple sclerosis, Disutility of relapse, Multi-attribute utility instrument, AQoL-8D, EQ-5D-5L-psychosocial, SF-6D, Cost–utility analysis, Health-related quality of life

## Abstract

**Background and aims:**

Relapses are an important clinical feature of multiple sclerosis (MS) that result in temporary negative changes in quality of life (QoL), measured by health state utilities (HSUs) (disutilities). We aimed to quantify disutilities of relapse in relapsing remitting MS (RRMS), secondary progressive MS (SPMS), and relapse onset MS [ROMS (including both RRMS and SPMS)] and examine these values by disability severity using four multi-attribute utility instruments (MAUIs).

**Methods:**

We estimated (crude and adjusted and stratified by disability severity) disutilities (representing the mean difference in HSUs of ‘relapse’ and ‘no relapse’ groups as well as ‘unsure’ and ‘no relapse’ groups) in RRMS (*n* = 1056), SPMS (*n* = 239), and ROMS (*n* = 1295) cohorts from the Australian MS Longitudinal Study’s 2020 QoL survey, using the EQ-5D-5L, AQoL-8D, EQ-5D-5L-Psychosocial, and SF-6D MAUIs.

**Results:**

Adjusted mean overall disutilities of relapse in RMSS/SPMS/ROMS were − 0.101/− 0.149/− 0.129 (EQ-5D-5L), − 0.092/− 0.167/− 0.113 (AQoL-8D), − 0.080/− 0.139/− 0.097 (EQ-5D-5L-Psychosocial), and − 0.116/− 0.161/− 0.130 (SF-6D), approximately 1.5 times higher in SPMS than in RRMS, in all MAUI. All estimates were statistically significant and/or clinically meaningful. Adjusted disutilities of RRMS and ROMS demonstrated a *U*-shaped relationship between relapse disutilities and disability severity. Relapse disutilities were higher in ‘severe’ disability than ‘mild’ and ‘moderate’ in the SPMS cohort.

**Conclusion:**

MS-related relapses are associated with substantial utility decrements. As the type and severity of MS influence disutility of relapse, the use of disability severity and MS-type-specific disutility inputs is recommended in future health economic evaluations of MS. Our study supports relapse management and prevention as major mechanisms to improve QoL in people with MS.

**Supplementary Information:**

The online version contains supplementary material available at 10.1007/s11136-023-03486-y.

## Introduction

Multiple sclerosis (MS) is an autoimmune/neurodegenerative disease in which the myelin sheath covering nerve fibers in the central nervous system (brain, optic nerves, and spinal cord) is damaged, leading to secondary axonal damage and neuronal death. This results in increasing disability due to impairments of cognitive, motor, and sensory functions, a substantial socioeconomic burden and lower individual health-related quality of life (HRQoL) over time [1, 2]. Approximately 85–90% of MS cases start as relapsing remitting MS (RRMS), with episodes of relapsing and remitting neurological dysfunction followed by partial or full recovery [3–5]. With time, the majority then enter a progressive phase of MS (characterized by an inexorable increase in disability) that is referred to as secondary progressive MS (SPMS) [3–6] Relapses (usually defined as episodes of new, worsening or recurring neurological symptoms, and disability lasting at least 24 h, preceded by at least a 30-day stability period for which there is no better explanation than MS) are one of the distinctive clinical features of RRMS and a challenging aspect of disease management for clinicians and patients [7, 8]. MS relapses generally result in worsening of MS symptoms for a period of up to several weeks, symptoms then recover either partially or fully over a period often up to 6 months [9]. Common symptoms/signs of MS-related relapse include weakness, numbness, or tingling, cognitive symptoms (e.g., memory, concentration, information processing, language), dizziness, balance, visual disturbance, and coordination problems. While continuous progression of disability without relapses/remissions is a defining feature of SPMS, transitioning between RRMS and SPMS is challenging to determine and people with SPMS can still experience relapses [6, 10, 11]. However, the frequency of experiencing a relapse event is generally shown to be lower in SPMS than in RRMS [12]. Because both the RRMS and SPMS start with acute relapse(s) and are the same continuum of disease, they can be combined to create an aggregate category of relapse onset MS (ROMS).

Those experiencing a relapse often need increased care and their health-related quality of life (HRQoL) is substantially impacted [13–15], which can be reflected as an overall weighted index of the health state utilities (HSUs [measuring the strength of preference for a given health state usually as a number between 0 = death and 1 = perfect health]) [16]. Multi-attribute utility instruments (MAUIs) such as the EQ-5D-3L or EQ-5D-5L [17, 18], Assessment of Quality of Life-8-Dimension (AQoL–8D) [19], EQ-5D-5L-Psychosocial [20], Short-Form-6-Dimension (SF-6D) versions 1 and 2 [21, 22], and others can be used to measure HSUs [23, 24] and are commonly used in health economic evaluation models to calculate quality-adjusted life-years (QALYs) [16]. QALYs are a measure that account for both the length and the quality of life and obtained by multiplying HSUs with survival time [2]. Temporary decrements in HSUs due to experiencing a MS-related relapse are often referred to as a “disutility” of relapse event or loss of utility due to relapse and can be measured by taking the difference between the mean HSUs of those with and those without the experience of relapse [13]. As relapses are significant predictors of lower HSU in people with MS [25], it is important to incorporate utility decrements due to relapses in economic evaluation models of MS subtypes to obtain precise estimates of QALYs when assessing the effectiveness and cost-effectiveness of various MS interventions.

While disutilities of MS relapse have been reported in the United States (US), Canada, and some European countries (Supplement 1), the estimates were predominantly obtained using the EQ-5D in overall samples of people, including multiple types of MS [12, 14, 26–35]. While a few studies report disutilities for RRMS cohorts only [36–41], there are a lack of relapse disutility estimates for SPMS, with only one US-based study reporting relapse disutilities separately for RRMS and SPMS, suggesting worse disutilities in SPMS than in RRMS [25]. Relapse disutility by level of disability was reported in a small number of studies [30, 32, 36], most of which classified the study participants into two broad Expanded Disability Status Scale (EDSS)-based disability categories (i.e., EDSS < 5 and EDSS ≥ 5), suggesting higher disutilities of relapse for those with an EDSS score of < 5. EDSS is widely used to quantify disability in MS and to monitor changes in the level of MS-related disability over time. It ranges from 0 to 10 in 0.5-unit increments, with higher scores representing higher levels of disability. Scoring is based on an examination by a neurologist [42]. These findings raise the question whether separate disutility inputs are needed for multi-state health economic models of RRMS, SPMS, and ROMS, requiring disability level-specific disutilities. MS-type-specific relapse disutilities for the severity categories of no (EDSS level: 0), mild (EDSS: 1–3.5), moderate (EDSS: 4.0–6.0), and severe (EDSS: 6.5–9.5) disability have not however been reported.

Against this backdrop, our study aimed to employ three common MAUIs (i.e., EQ-5D-5L, SF-6D, and AQoL-8D) as well as the new, validated EQ-5D-5L-Psychosocial that addresses the psychosocial gaps in the EQ-5D-5L by including four bolt-on questions from the AQoL-8D regarding vitality, relationships, sleep, and social isolation [20, 43] to quantify disutilities of relapse in the total sample and disability severity-specific samples of people with RRMS, SPMS, and ROMS. Additionally, we aimed to identify patient subgroups that are more susceptible to the negative utility impacts of relapses and to generate a database of MS type and disability severity-specific relapse disutilities to be incorporated in the multi-state health economic evaluation models of RRMS, SPMS, and ROMS.

## Materials and methods

### Study design

**Fig. 1:**
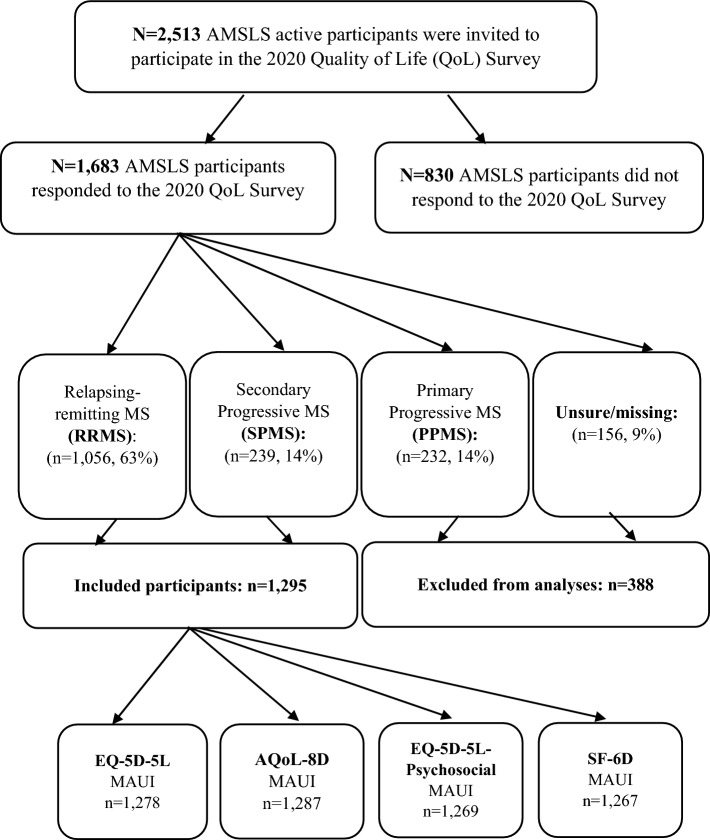
Flow of Australian MS Longitudinal Study (AMSLS) participants into the study *Note*: *MS* multiple sclerosis; *RRMS* relapsing remitting MS; *SPMS* secondary progressive MS; *PPMS* primary progressive MS; *AQoL*-*8D* assessment of quality of life-8-dimension; *EQ*-*5D*-*5L*-*Psychosocial*; and *SF*-*6D* short-form-6-dimension

The Australian Multiple Sclerosis Longitudinal Study (AMSLS) is a large national sample of Australians with MS that has been shown to be representative [44]. Australian residents, minimum 18 years of age with a diagnosis of MS, are eligible to join. Ethics approval for the study was obtained from the University of Tasmania’s Human Research Ethics Committee (ref: H0014183), and written informed consent was received from all AMSLS participants. Between July 31 and September 30, 2020, *n* = 2513 active AMSLS participants were invited to complete the 2020 Quality of Life (QoL) survey, with *n* = 1683 (67%) participants responding (Fig. 1). Of these, *n* = 1056 and *n* = 239 were identified as RRMS and SPMS, respectively. We combined RRMS and SPMS as ROMS (*n* = 1295) (Fig. 1).

Participants reported their AQoL-8D, EQ-5D-5L, and Short-Form Survey (SF)-36 profiles by completing the individual MAUIs contained in the 2020 QoL Survey. The choice of MAUIs in the 2020 QoL Survey was based on our findings from previous work, suggesting EQ-5D, SF-6D, and AQoL suite of instruments being the dominant elicitation instruments for HSUVs in MS in Australia [45]. The order of presenting the MAUI questionnaires was randomized. The 2020 QoL survey also captured data on participants’ clinical and sociodemographic characteristics including their age, sex, State/Territory of usual residence, disability severity (see below), disease course, and disease-modifying therapy (DMT) use at the time of survey. MS duration since diagnosis and education levels were obtained from a previous survey.

### Assessment of relapses

Herein this study, a relapse of MS was defined as the development of new symptom(s) or worsening of old symptom(s) lasting longer than 48 h. Notably, the change in symptoms could not be due to heat or illness (e.g., flu, cold, or urinary tract infection). Based on this definition, participants were asked to report if, at the time of completing the survey, they were currently experiencing ongoing symptoms due to a relapse, with the answer choices of ‘Yes,’ ‘No,’ and ‘Unsure.’ Throughout the rest of this paper, those answering ‘Yes,’ ‘No,’ and ‘Unsure’ to this question are referred to as ‘relapse,’ ‘no relapse,’ and ‘unsure’ groups, respectively.

### Assessment of disutility associated with MS relapse

Disutility generally represents the decrement in HSU due to a particular symptom or complication and may be obtained by subtracting HSU for a health state which includes the relevant component from a health state that is identical except for the absence of that component [46]. HSUs in our study were generated using four generic MAUIs [i.e., the EQ-5D-5L, AQoL-8D, EQ-5D-5L-Psychosocial, and the SF-6D version 1 (v1))] based on preference weights derived from the Australian general population [18, 20, 47, 48]. Disutilities of relapse were then obtained by calculating the mean differences in HSUs of those experiencing symptom(s) of relapse at the time of survey completion and those without; hence, in the present context, relapse disutility represents a decrement in the mean HSU of ‘relapse group’ because of experiencing symptoms of a relapse event.

The EQ‐5D-5L asks participants to indicate problems on a five-level scale for each of the five health dimensions (i.e., mobility, self‐care, usual activities, pain/discomfort, and anxiety/depression) and describes 3,125 possible health states [49]. The EQ-5D-5L HSUs were elicited using preference weights for different health states developed with the Australian general population [18]. The AQoL-8D comprised 35 items that loaded to three individual dimensions (independent living, senses, and pain) of physical and five (mental health, self-worth, relationships, coping, and happiness) of psychosocial health [50]. We combined the individual dimension scores according to the AQoL-8D’s utility algorithm to generate HSUs for our sample. [50]

The EQ-5D-5L-Psychosocial is a nine-item instrument which adopts the EQ-5D-5L and four psychosocial bolt-on questions from the AQoL-8D regarding vitality (AQoL-8D Question 1), community connectedness (AQoL-8D Question 10), sleep (AQoL-8D Question 12), and social isolation (AQoL-8D Question 31) [20, 43]. Finally, the SF-36 is a generic HRQoL instrument comprising 36 items. We converted SF-36 responses into a six-dimensional preference-based MAUI (the SF-6D Version 1 [v1]) according to the algorithm developed by Brazier et al. 2002 [48]. We then applied Australian SF-6D preference weights to each level in each SF-6D dimension (i.e., physical functioning, role limitations, social functioning, pain, mental health, and vitality) to calculate utilities [47]. The algorithmic ranges of the HSUs for the four instruments’ Australian value sets are -0.68 to 1.00 for EQ-5D-5L, 0.046 to 1.00 for EQ-5D-5L-Psychosocial, 0.09 to 1.00 for AQoL-8D, and -0.35 to 1.00 for SF-6Dv1. Comparisons of the dimensions and content of the four MAUIs are provided by Supplement 2.

### Measurement of disability

We measured disability using the Patient-Determined Disease Steps (PDDS) scale, a validated patient-reported outcome of mobility-based functional disability in MS. A high positive correlation has been reported between PDDS and the Expanded Disability Status Scale (EDSS) scores [29, 42, 51–54]. Following previous publications [29, 55–59], we converted PDDS scores into their EDSS equivalents and grouped the study participants into four broad disability categories: no disability (EDSS level: 0), mild disability (EDSS 1–3.5), moderate disability (EDSS 4–6), and severe disability (EDSS 6.5–9.5) (Supplement 3).

### Statistical analyses

We used descriptive analysis to quantitatively summarize the respondents’ clinical and sociodemographic characteristics in the RRMS, SPMS and in the total (ROMS) samples. We then compared the characteristics of respondents with and without a current relapse in each of the three MS cohorts using *t* test (for continuous variables) and *χ*^*2*^ test (for categorical variables). We also compared the characteristics of the 2020 QoL survey’s respondents with non-respondents to assess study sample’s representativeness.

Relapse disutilities were estimated by calculating the crude and adjusted (for the confounders of age, disease duration since diagnosis, education level, and disability severity) mean [95% confidence intervals (CIs))] differences in HSUs of ‘relapse’ and ‘no relapse’ groups, as well as ‘unsure’ and ‘relapse’ groups in the three MS cohorts using univariable and multivariable linear regression models. Relapse disutilities by disability severity were then evaluated by taking the mean difference in the disability severity stratified (crude and adjusted) HSUs of ‘relapse’ and ‘no relapse’ groups in the three MS cohorts. Confounders were identified based on their significant association with current relapse status and HSUs. The choice of confounders included in the models was also supported by the relevant existing literature assessing HRQoL in MS [5, 58, 59].

All analyses were performed using STATA/IC for Windows (version 17.0; Stata Corp LP, College Station, TX, USA). Disutilities of relapse were evaluated based on both statistical significance and clinical importance. Statistical significance was set as a *P*-value ≤ 0.05 (two-tailed). Whereas, disutilities were considered clinically important if they met or exceeded the minimum clinically important difference (MID) thresholds of 0.052 for the EQ-5D-5L [60], 0.06 for the AQoL-8D [61], and 0.041 [62] for the SF-6D. We also adopted the MID threshold of the AQoL-8D (0.06) for the EQ-5D-5L-Psychosocial MAUI given that the new MAUI has been validated for our AMSLS cohort and displayed interchangeability with the AQoL-8D [43].

## Results

### Study sample’s clinical and sociodemographic features

A summary of participants’ flow into the study is provided by Fig. 1, including the number of participants for whom disutilities of relapse could be generated using each of the four MAUIs mentioned above. We compared respondents (*n* = 1683) with non-respondents (*n* = 830) and found that respondents were largely representative of the AMSLS sample as evidenced by similar sex ratios, education levels, and State/Territory distributions. However, respondents were slightly older (+ 3 years) and had disease duration since diagnosis longer by 2 years (Supplement 4). Of the 1,683 respondents, more than three-quarters (*n* = 1295) had either RRMS (*n* = 1056, 82%) or SPMS (*n* = 239, 18%) and were included in the current analyses. HSUs were generated for approximately 99% of participants for the EQ-5D-5L (*n* = 1278), AQoL-8D (1,287), EQ-5D-5L-Psychosocial (*n* = 1269), and SF-6Dv1 (*n* = 1267) (Fig. 1).

Table 1 provides characteristics of the overall RRMS, SPMS, and ROMS cohorts and by current relapse status. When we compared participants with RRMS to SPMS, as expected, we found that the SPMS cohort was older (62.8 years vs 55.7 years), had longer disease duration (22.6 years vs 17.5 years), had a higher proportion of people in moderate-to-severe disability categories (92% vs 40%), and a smaller percentage (42% vs 60%) using DMTs. Of the RRMS and SPMS cohorts, *n* = 132 (13%) and *n* = 46 (19%) reported a current relapse event, respectively. When we compared people with current relapse with those without in the RRMS cohort (Table 1), we found significant differences in education levels, disability severity levels, and mean MS duration since diagnosis. For instance, 42% of ‘no relapse’ group had university degrees, compared to 30% in ‘relapse’ group. Sixty-nine percent of ‘relapse’ group participants were living with moderate-to-severe disability, compared to 33% in ‘no relapse’ group. However, no differences were found in age, sex, or DMT use. When we repeated this for the SPMS cohort, we found no differences between the ‘relapse’ and ‘no relapse’ groups in any of Table 1 characteristics. Table 1 also compared ‘relapse’ and ‘no relapse’ groups in the total (ROMS) cohort, reporting similar results to those in the RRMS cohort.

**Table 1:** Clinical and sociodemographic characteristics of respondents by MS type and current history of relapse

Characteristics#	RRMS					SPMS					ROMS (RRMS + SPMS)				
	All (*n* = 1056)	Currently experiencing a relapse			*P* value*	all (*n* = 239)	Currently experiencing a relapse			*P* value*	all (*n* = 1,295)	Currently experiencing a relapse			*P* value*
		No (*n* = 835, 79.1%)	Yes (*n* = 132, 12.5%)	Unsure (*n* = 85, 8.0%)			No (*n* = 149, 62.3%)	Yes (*n* = 46, 19.2%)	Unsure (n = 35, 14.6%)			No (*n* = 984, 76.0%)	Yes (*n* = 178, 13.7%)	Unsure (*n* = 120, 9.3%)	
Age, average in years	55.7	55.5	56.5	56.5	0.318	62.8	62.2	63.4	62.8	0.479	57.02	56.5	58.3	58.3	** < 0.024**
Sex															
Male	15 (161)	15 (128)	14 (19)	16 (14)		23 (55)	24 (35)	22 (10)	20 (7)		17 (216)	17 (163)	16 (29)	17 (21)	
Female	85 (895)	85 (707)	86 (113)	84 (71)	0.781	77 (184)	76 (114)	78 (36)	80 (28)	0.805	83 (1,079)	83 (821)	84 (149)	83 (99)	0.928
Education level															
Primary and secondary school	21 (220)	20 (167)	25 (33)	22 (19)		22 (52)	20 (30)	22 (10)	29 (10)		21 (272)	20 (197)	24 (43)	24 (24)	
Occupational certificate or diploma	33 (352)	32 (267)	40 (53)	35 (30)		35 (83)	32 (48)	39 (18)	34 (12)		34 (435)	32 (315)	40 (71)	35 (42)	
University bachelor’s degree	22 (231)	24 (197)	13 (17)	19(16)		20 (48)	22 (32)	22 (10)	17(6)		21 (279)	23 (229)	15 (27)	18 (22)	
University postgraduate degree	18 (190)	18 (152)	17 (22)	19 (16)		16 (39)	17 (26)	13 (6)	17 (6)		18 (229)	18 (178)	16 (28)	18 (22)	
Other/Missing	3 (36)	4 (33)	**	**		4 (10)	5 (8)	**	**		4 (46)	4 (41)	**	**	
Missing	3 (27)	2 (19)	5 (6)	**	**0.016**	3 (7)	3 (5)	**	**	0.864	3 (34)	2 (24)	4 (7)	**	**0.026**
DMT Usage Status															
Yes	60 (634)	62 (515)	61(80)	45 (38)		42 (101)	42 (63)	37(17)	51 (18)		57 (735)	59 (578)	55 (97)	46 (56)	
No	19 (203)	20 (166)	15 (20)	19 (16)		39 (92)	41 (61)	39 (18)	23 (8)		23 (295)	23 (227)	21 (38)	20 (24)	
Missing	21 (219)	18 (154)	24 (32)	36 (31)	0.188	19 (46)	17 (25)	24 (11)	26 (9)	0.538	20 (265)	18 (179)	24 (43)	33 (40)	0.176
Disability severity															
No disability	32 (343)	38 (321)	8 (10)	14 (12)		**	**	**	**		27 (346)	33 (324)	6 (10)	10 (12)	
Mild disability	27 (281)	28 (235)	23 (31)	16 (14)		5 (12)	4 (6)	7 (3)	6 (2)		23 (293)	24 (241)	19 (34)	13 (16)	
Moderate disability	35 (368)	28 (235)	62 (82)	58 (49)		39 (94)	39 (58)	48 (22)	40 (14)		36 (462)	30 (293)	58 (104)	52 (63)	
Severe disability	5 (58)	5 (40)	7 (9)	9 (8)		53 (126)	53 (79)	43(20)	54 (19)		14 (184)	12 (119)	16 (29)	23 (27)	
Missing	< 1 (6)	**	**	**	** < 0.001**	**	**	**	**	0.607	< 1 (10)	< 1 (7)	**	**	** < 0.001**
MS duration since diagnosis, average in years	17.5	17.4	18.9	17.1	**0.038**	22.6	22.7	23.3	21.1	0.735	18.5	18.3	20.1	18.3	**0.007**

Bold values indicate the statistical significance

^**#**^All data were reported in % (n) unless specifically mentioned

*P-values are based on Pearson Chi-square test and *t* test and compared the characteristics of respondents currently experiencing a relapse with those not currently experiencing a relapse. **Observations with a sample size of less than 5 are suppressed

*MS* multiple sclerosis; *RRMS* relapsing remitting MS; *SPMS* secondary progressive MS; *ROMS* relapse onset MS; *DMT* disease-modifying therapy

### Mean disutilities by MS subgroups

Table 2 provides the adjusted mean (95% CI) EQ-5D-5L, AQoL-8D, EQ-5D-5L-Psychosocial, and SF-6Dv1 relapse disutilities for the three MS subgroups. Table 2 shows that in those with RRMS, compared to those not experiencing a current relapse, those experiencing a current relapse had considerably lower utility scores for all MAUIs, with the mean EQ-5D-5L, AQoL-8D, EQ-5D-5L-Psychosocial, and SF-6Dv1 disutilities of − 0.101, − 0.092, − 0.080, and − 0.116, respectively. Mean disutilities for participants with SPMS were − 0.149 for the EQ-5D-5L, − 0.167 for the AQoL-8D, − 0.139 for the EQ-5D-5L-Psychosocial, and − 0.161 for the SF-6Dv1, approximately 1.5 times higher than those of participants with RRMS, regardless of the choice of MAUI. When we examined adjusted relapse disutilities of participants with ROMS, they were similar to those of participants with RRMS for all MAUIs. Adjusted mean relapse disutilities were statistically significant and clinically important for RRMS, SPMS, and ROMS, regardless of the MAUI choice.

**Table 2:** Adjusted mean (95% confidence interval [CI]) disutilities of current relapse in people with RRMS, SPMS, and ROMS

	RRMS (*n* = 1056)*		SPMS (*n* = 239)*		ROMS (*n* = 1295) *	
	Mean (95%CI)	*P* value	Mean (95%CI)	*P* value	Mean (95%CI)	*P* value
EQ-5D-5L						
No			0.00 (Ref.)			
Yes	**− 0.101 (− 0.140, − 0.061)**	** < 0.001**	**− 0.149 (− 0.257, − 0.041)**	**0.006**	**− 0.129 (− 0.167, − 0.091)**	** < 0.001**
Unsure	**− 0.114 (− 0.161, − 0.067)**	** < 0.001**	**− 0.153 (− 0.273, − 0.033)**	**0.011**	**− 0.128 (-0.172, − 0.083)**	** < 0.001**
AQoL-8D						
No			0.00 (Ref.)			
Yes	**− 0.092 (− 0.127, − 0.057)**	** < 0.001**	**− 0.167 (− 0.228, − 0.105)**	** < 0.001**	**− 0.113 (− 0.143, − 0.083)**	** < 0.001**
Unsure	**− 0.061 (− 0.102, − 0.019)**	**0.004**	**− 0.099 (− 0.167, − 0.031)**	**0.005**	**− 0.067 (− 0.103, − 0.032)**	** < 0.001**
EQ-5D-5L-Psychosocial						
No			0.00 (Ref.)			
Yes	**− 0.080 (− 0.112, − 0.047)**	** < 0.001**	**− 0.139 (− 0.199, − 0.080)**	** < 0.001**	**− 0.097 (− 0.126, − 0.069)**	** < 0.001**
Unsure	**− 0.076 (− 0.114, − 0.038)**	** < 0.001**	**− 0.098 (− 0.164, − 0.032)**	**0.005**	**− 0.082 (− 0.115, − 0.049)**	** < 0.001**
SF-6D						
No			0.00 (Ref.)			
Yes	**− 0.116 (− 0.162, − 0.069)**	** < 0.001**	**− 0.161 (− 0.249, − 0.075)**	** < 0.001**	**− 0.130 (− 0.171, − 0.089)**	** < 0.001**
Unsure	**− 0.091 (− 0.146, − 0.036)**	**0.001**	− 0.080 (− 0.177, 0.016)	0.086	**− 0.082 (− 0.130, − 0.034)**	**0.001**

Bold values indicate the statistical significance

*MS* multiple sclerosis; *RRMS* relapsing remitting MS; *SPMS* secondary progressive MS; *ROMS* relapse onset MS (which includes both RRMS and SPMS)

*Adjusted for age, disease severity, education levels, and disease duration

Table 2 also reports disutility values by MAUI and MS type for those unsure about their current relapse status, suggesting that the adjusted disutilities for this group of patients were generally lower than those who reported the current experience of relapse. Interestingly, the EQ-5D-5L-adjusted disutilities were similar for the unsure and relapse groups compared to those lower adjusted disutilities for the unsure group reported for the AQoL-8D, EQ-5D-5L-Psychosocial, and SF-6Dv1. Supplement 5 reports unadjusted disutilities by MS type for the four MAUIs.

### Mean disutilities by MS subgroups and disability severity

**Fig. 2:**
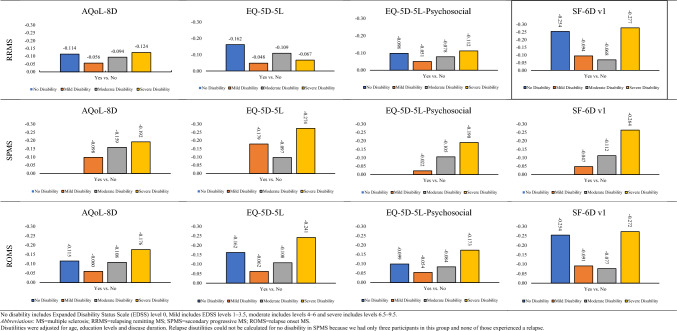
Adjusted disutilities of relapse in people with RRMS, SPMS, and ROMS, by disability severity. No disability includes Expanded Disability Status Scale (EDSS) level 0, Mild includes EDSS levels 1–3.5, moderate includes levels 4–6, and severe includes levels 6.5–9.5. *MS* multiple sclerosis; *RRMS* relapsing remitting MS; *SPMS* secondary progressive MS; *ROMS* relapse onset MS. Disutilities were adjusted for age, education levels, and disease duration. Relapse disutilities could not be calculated for no disability in SPMS because we had only three participants in this group and none of those experienced a relapse

Table 3 and Fig. 2 report adjusted EQ-5D-5L, AQoL-8D, EQ-5D-5L-Psychosocial, and SF-6Dv1 relapse disutilities by disability severity in the three MS cohorts. When we compared the ‘relapse’ with ‘no relapse’ groups, adjusted mean disutilities of the RRMS cohort first decreased and then increased with increasing disability severity, demonstrating a *U*-shaped relationship between relapse disutilities and disability severity (Fig. 2). This *U*-shaped relationship was particularly apparent with the AQoL-8D (− 0.114, no disability; − 0.056, mild disability; − 0.094, moderate disability; and − 0.124, severe disability), EQ-5D-5L-Psychosocial (− 0.098, no disability; − 0.051 mild disability; − 0.078, moderate disability; and − 0.112, severe disability), and the SF-6D (no disability − 0.254; mild disability − 0.094; moderate disability − 0.068; and severe disability − 0.277). With the EQ-5D-5L, the relapse disutility was highest (− 0.162) for participants with no disability, which substantially decreased when moving from no disability to mild disability and then remained similar across the mild, moderate, and severe disability subgroups (Fig. 2). Except for the mild disability group, the AQoL-8D and SF-6Dv1-adjusted disutilities were statistically significant for all disability groups for the RRMS cohort (Table 3). However, the adjusted EQ-5D-5L and EQ-5D-5L-Psychosocial disutilities were statistically insignificant for mild as well as severe disability groups. Disability severity-specific relapse disutilities of the RRMS cohort were clinically important for all disability groups, except for mild disability category of EQ-5D-5L, AQoL-8D, and EQ-5D-5L-Psychosocial MUIs.

**Table 3:** Adjusted mean (95% confidence intervals) disutilities of relapse in RRMS, SPMS, and ROMS, by disability severity

	Disability severity			
	No disability	Mild disability	Moderate disability	Severe disability
RRMS*				
EQ-5D-5L	*n* = *337*	*n* = *280*	*n* = *365*	*n* = *58*
No	0.00 (Ref.)			
Yes	**− 0.162 (− 0.258, − 0.067)**	− 0.048 (− 0.127, 0.031)	**− 0.109 (− 0.165, − 0.053)**	− 0.067 (− 0.299, 0.165)
Unsure	**− 0.156 (− 0.244, − 0.068)**	**− 0.153 (− 0.258, − 0.047)**	− 0.059(− 0.131, 0.012)	− 0.195 (− 0.437, 0.046)
AQoL-8D	*n* = *342*	*n* = *281*	*n* = *365*	*n* = *57*
No	0.00 (Ref.)			
Yes	**− 0.114 (− 0.212, − 0.017)**	− 0.056 (− 0.128, 0.014)	**− 0.094 (− 0.143, − 0.044)**	**− 0.124 (− 0.260, 0.012)**
Unsure	**− 0.121 (− 0.210, − 0.031)**	− 0.080 (− 0.176, 0.015)	− 0.003 (− 0.063, 0.063)	**− 0.149 (− 0.291, − 0.008)**
EQ-5D-5L-Psy	*n* = *335*	*n* = *279*	*n* = *360*	*n* = *57*
No	0.00 (Ref.)			
Yes	**− 0.098 (− 0.190, − 0.006)**	− 0.051 (− 0.117, 0.015)	**− 0.078 (− 0.123, − 0.033)**	− 0.112 (− 0.238, 0.015)
Unsure	**− 0.126 (− 0.210, − 0.041)**	− 0.128 (− 0.217, 0.039)	− 0.019 (− 0.075, 0.037)	− 0.098 (− 0.229, 0.032)
SF-6D	*n* = *341*	*n* = *274*	*n* = *360*	*n* = *57*
No	0.00 (Ref.)			
Yes	**− 0.254 (− 0.381, − 0.127)**	− 0.094 (− 0.193, 0.004)	**− 0.068 (− 0.133, − 0.034)**	**− 0.277 (− 0.442, − 0.111)**
Unsure	**− 0.194 (− 0.310, − 0.078)**	**− 0.172 (− 0.302, − 0.041)**	0.016 (− 0.066, 0.099)	**− 0.209 (− 0.379, − 0.037)**
SPMS*				
EQ-5D-5L	*n* = *2*	*n* = *12*	*n* = *92*	*n* = *123*
No	0.00 (Ref.)			
Yes	*NA*^	− 0.179 (− 0.493, 0.134)	− 0.097 (− 0.210, 0.016)	**− 0.274 (− 0.409, − 0.139)**
Unsure	*NA*^	− 0.439 (− 1.011, 0.134)	− 0.024 (− 0.157, 0.108)	**− 0.243 (− 0.383, − 0.103)**
AQoL-8D	*n* = *3*	*n* = *12*	*n* = *94*	*n* = *124*
No	0.00 (Ref.)			
Yes	*NA*^	− 0.098 (− 0.387, 0.190)	**− 0.159 (− 0.244, − 0.074)**	**− 0.192 (− 0.282, − 0.102)**
Unsure	*NA*^	− 0.197 (− 0.547, 0.152)	− 0.045 (− 0.147, 0.056)	**− 0.130 (− 0.222, − 0.0383)**
EQ-5D-5L-Psy	*n* = *2*	*n* = *12*	*n* = *92*	*n* = *123*
No	0.00 (Ref.)			
Yes	*NA*^	− 0.022 (− 0.343, 0.299)	**− 0.105 (− 0.187, − 0.023)**	**− 0.190 (− 0.275, − 0.105)**
Unsure	*NA*^	− 0.282 (− 0.834, 0.269)	− 0.006 (− 0.112, 0.080)	**− 0.156 (− 0.245, − 0.067)**
SF-6D	*n* = *3*	*n* = *11*	*n* = *92*	*n* = *121*
No	0.00 (Ref.)			
Yes	*NA*^	− 0.047 (− 0.521, 0.426)	**− 0.112 (− 0.220, − 0.005)**	**− 0.264 (− 0.401, − 0.127)**
Unsure	*NA* ^	0.137 (− 0.422, 0.697)	− 0.112 (− 0.240, 0.016)	− 0.059 (− 0.196, 0.079)
ROMS*				
EQ-5D-5L	*n* = *339*	*n* = *292*	*n* = *457*	*n* = *181*
No	0.00 (Ref.)			
Yes	**− 0.162 (− 0.258, − 0.067)**	− 0.062 (− 0.139, 0.015)	**− 0.108 (− 0.160, − 0.056)**	**− 0.241 (− 0.363, − 0.118)**
Unsure	**− 0.156 (− 0.244, − 0.069)**	**− 0.212 (− 0.316, − 0.107)**	**− 0.065 (− 0.130, − 0.001**)	**− 0.204 (− 0.333, − 0.074)**
AQoL-8D	*n* = *345*	*n* = *293*	*n* = *459*	*n* = *181*
No	0.00 (Ref.)			
Yes	**− 0.115 (− 0.212, − 0.017)**	− 0.060 (− 0.127, 0.007)	**− 0.108 (− 0.152, − 0.065)**	**− 0.176 (− 0.250, − 0.102)**
Unsure	**− 0.121 (− 0.211, − 0.032)**	**− 0.109 (− 0.201, − 0.017)**	− 0.009 (− 0.063, 0.046)	**− 0.124 (− 0.201, − 0.047)**
EQ-5D-5L-Psy	*n* = *337*	*n* = *291*	*n* = *452*	*n* = *180*
No	0.00 (Ref.)			
Yes	**− 0.099 (− 0.191, − 0.070)**	− 0.054 (− 0.117, 0.008)	**− 0.084 (− 0.124, − 0.044)**	**− 0.173 (− 0.244, − 0.102)**
Unsure	**− 0.126 (− 0.211, − 0.042)**	**− 0.152 (− 0.238, − 0.068)**	− 0.021 (− 0.070, 0.028)	**− 0.129 (− 0.204, − 0.054)**
SF-6D	*n* = *344*	*n* = *285*	*n* = *452*	*n* = *178*
No	0.00 (Ref.)			
Yes	**− 0.254 (− 0.381, − 0.128)**	− 0.091 (− 0.184, 0.001)	**− 0.077 (− 0.134, − 0.020)**	**− 0.272 (− 0.380, − 0.164)**
Unsure	**− 0.194 (− 0.310, − 0.078)**	**− 0.170 (− 0.300, − 0.041)**	− 0.020 (− 0.091, 0.051)	− 0.080 (− 0.191, 0.031)

Bold values indicate the statistical significance

*MS* multiple sclerosis; *RRMS* relapsing remitting MS; *SPMS* secondary progressive MS; *ROMS* relapse onset MS (which includes both RRMS and SPMS. EQ-5D-5L-Psy = EQ-5D-5L-Psychosocial; *NA* not available

^Disutility coefficient could not be estimated as *n* = 0 participants had relapse in this category

*Adjusted for age, education levels, and disease duration

No disability includes Expanded Disability Status Scale (EDSS) level 0, Mild includes EDSS levels 1–3.5, moderate includes levels 4–6, and severe includes levels 6.5–9.5

The mean EQ-5D-5L, AQoL-8D, EQ-5D-5L-Psychosocial, and SF-6Dv1 disutilities of the SPMS sample could not be calculated for the no disability group due to low numbers **(**Table 3**)**. While disutilities for mild disability were available, however, coefficients were statistically insignificant owing to low sample numbers (up to 12) in this group (Table 3). Moderate disability category of the SPMS cohort contained a sufficiently large number of participants (*n* = 94) and disutility values for this group of participants were significant for the AQoL-8D, EQ-5D-5L-Psychosocial, and SF-6D. Finally, the relapse disutility for people with severe MS-related disability and SPMS were large and statistically significant, namely − 0.274 for the EQ-5D-5L; − 0.192 for the AQoL-8D; − 0.190 for the EQ-5D-5L-Psychosocial; and − 0.264 for SF-6D (Table 3). Overall, we observed an increasing trend of relapse disutilities from ‘mild’ to ‘severe’ disability in the SPMS cohort (Fig. 2). Relapse disutilities of the SPMS cohort were clinically important for moderate and severe disability groups in all MAUIs.

Table 3 and Fig. 2 also compared ‘relapse’ and ‘no relapse’ groups according to the categories of disability in the total (ROMS) cohort and found similar but expected results to those in the RRMS cohort. We also compared the ‘unsure’ and ‘no relapse’ groups according to the categories of disability in participants with ROMS, RRMS, and SPMS (Table 3). Our results demonstrated that disutilities of ‘unsure’ group were generally lower than those of ‘relapse’ group. However, these estimates were not entirely reliable due to small sample limitations. Supplement 6 reports unadjusted disutilities by MS type and disability severity for the four MAUIs.

## Discussion

Our study provides a comprehensive assessment of the overall and disability severity-specific disutilities of relapse in a large sample of Australians with RRMS, SPMS, and ROMS, using three commonly used MAUIs (i.e., EQ-5D-5L, AQoL-8D, and SF-6D) and the new, validated EQ-5D-5L-Psychosocial instrument. Our estimates of relapse disutility are adjusted for the confounders of age, disease duration since diagnosis, and other factors to account for their impact on reported results. PPMS cases cannot be included in the analyses as they experience neurological worsening from the onset without relapses and hence, relapse or disutility of relapse are not relevant for this group of people MS. We found that MS-related relapses result in statistically significant and/or clinically important HRQoL decrements (disutilities) that differed between MS subtypes, with SPMS attracting an overall mean relapse disutility of approximately 1.5 times higher than that of RRMS, regardless of the choice of MAUI. Our results demonstrated that disutilities of ‘unsure’ group were generally lower than those of ‘relapse’ group, which is as expected from a group who must have felt some worsening, but they were not entirely sure whether it could be classified as a relapse. Relapse disutilities also differed by participants’ disability levels, with no disability and severe disability having higher mean disutilities than mild and moderate disability. These findings suggest that both the type of MS and level of disability influence disutility of relapse; hence, future health economic evaluations of MS should utilize the disability severity- and MS-type-specific disutility inputs instead of relying on mean disutility values derived from an overall sample of people with more than one type of MS at varying levels of disability. Furthermore, the optimal management and/or prevention of MS relapses, particularly in those with no disability and severe disability, may substantially help in maintaining HRQoL for people living with MS.

Based on our findings the overall mean disutilities of RRMS and ROMS cohorts ranged between − 0.080 and − 0.130, which aligns with previous findings from Europe and other nations [12, 27, 28, 32–35, 39, 41]. Mean disutilities of SPMS cohort in our study ranged between 0.139 and 0.167. There are a lack of research studies reporting the relapse disutilities in SPMS cohorts. However, one United States-based study reported relapse disutilities for RRMS and SPMS cohorts separately [25], suggesting worse overall mean disutilities in SPMS than in RRMS, which also accords with the findings of our study. While the exact rationale behind higher relapse disutilities in SPMS compared to RRMS is unknown, it may be driven through the fact that a significant majority of people with SPMS fall within the severe disability category. Here, a change in disability severity can have a marked effect on mobility. For instance, a change of 1 EDSS point in those with an EDSS of 6 results in moving from using a single walking aid (crutch or stick) to being largely confined to a wheelchair or from 8 to 9 results in a change from confined to a wheelchair to confined to bed. In turn, the MS-related severe disability category can associate with higher relapse disutilities as reported in Table 3 of our study and explored further in the next paragraph.

We found a U-shaped relationship between relapse disutilities and MS-related disability severity, as evidenced by worse disutility estimates for those with no disability and severe disability compared to those with mild and moderate disability. This could be explained by the relative HRQoL sensitivity for people with MS who are classified with ‘no’ or ‘severe’ disability status. Specifically, at the outset of their disease course, people with MS are not familiar with the HRQoL changes that occur with a MS-related relapse. In turn, we suggest that they are considerably impacted with these relapses early in their disease course. However, as the chronic and complex disease course of MS progresses (from mild to moderate disability levels), people living with MS may learn and adapt to these relapse events and their concomitant sensitivity to these relapse events reduces. Moreover, when in the severe disability category, the impact of relapse events intensifies again, and because of the worst health state, people with MS are likely to be substantially impacted by relapse events during this phase of the disease course. Existing literature explores how and why people’s perceptions of their health may differ and change over time, particularly among those who experience a long-term health conditions, such as MS. These changes arise due to processes such as “adaptation,” as people become increasingly accustomed to living in a compromised health state [63] and “shifting inter-personal and intra-personal comparisons” as they encounter more serious health states in themselves and others over time [64]. Therefore, several mechanisms other than what has been hypothesized above may also be at play in creating differences in utilities within and between respondents (or groups of respondents) in our sample. The rationale of *U*-shaped relationship between disutilities and MS-related relapse is also supported under the Hedonic Psychology research, which studies determinants of well-being and the impact of judgmental processes involved in reports of well-being [65].

Our study has used published fixed minimal clinically important difference (MCID) cut-offs for each instrument for all groups of people with MS included in our study, with any reductions in disutility values considered clinically important if they met or exceeded the relevant MCID thresholds. While any clinically important changes in utility scores of a patient (or a group of patients) may suggest a change in patient’s clinical management is necessary to ensure its consistency with patient’s updated health status, our study is not aimed at exploring what clinical impact these changes will have on patients, as the clinical impact of these changes will vary between patients by the severity of their illness, their sociodemographic features (for example, their age, and social status), their baseline health status, their impacted domain(s) of health, and their own concepts of health and improvement [66].

Some previous studies investigated the relationship between relapse disutilities and disability severity; however, their disability categories did not match our disability categories. To illustrate, a Canadian study identified a decreasing trend of utility loss with an EDSS increase (i.e., 0.10 utility loss for EDSS 1–2; 0.05 utility loss for EDSS 3–4; and 0.05 utility loss for EDSS 5–6) [36]; however, this study did not investigate people with MS in the severe disability category. A German study of 2793 participants reported an overall mean utility loss of 0.10, with 0.09 for EDSS < 5 and 0.05 for EDSS ≥ 5 [32]. Despite the inclusion of people with all EDSS levels, this study did not report disutility estimates for more granular (‘no’, ‘mild’, ‘moderate,’ and ‘severe’) categories of disability severity.

Our findings are in line with previous evidence and suggest that MS-related relapses are associated with substantial HSU decrements that vary by the type of MS and disability categories of people with MS. Therefore, cost-effective interventions to prevent and/or optimally manage MS-related relapses are important to ensure better health outcomes, particularly for those who have SPMS, and those people with MS living with no disability or severe disability. An important finding for health economic model inputs was that disability severity classification and MS type in terms of relapse disutility are sensitive discriminators. Therefore, the use of disability severity and MS-type-specific disutility input parameters in future multi-state health economic models of MS is important to facilitate the efficient allocation of scarce healthcare resources by minimizing the uncertainty in identifying interventions that are best value for money.

There are a couple of unexpected results in Table 1. For example, when we compared people with current relapse with those without, statistically significant differences in education levels between ‘relapse’ and ‘no relapse’ groups were found. However, no differences were found on the rate of DMT usage between the two groups. This may give rise to questions as to why relapse rates would differ according to level of education. Additionally, we expect relapse rates among those using DMTs to be lower. A possible explanation could be the existence of treatment bias and those with higher relapse rates are given treatment which does not absolutely eliminate relapses. Also, better educated people are more likely to be on therapy [68]. While there could be several other justifications to support these unexpected results, we suggest no casual inferences should be drawn from the results in Table 1 as these results are based on the Chi-squared test, which does not provide a suitable basis for conclusions regarding the nature and strength of association between education or DMTs usage and relapse rate [67].

An important strength of our study is that results are derived from a large sample that has been shown to be representative, with a sufficient number of RRMS and SPMS cases for relapse disutility analyses by MS type. Also, we used four MAUIs for disutility assessment including the well-validated preferentially sensitive and detailed AQoL-8D and the new EQ-5D-5L-Psychosocial that has been previously validated in our AMSLS cohort and found to be interchangeable with the AQoL-8D with reduced participant burden (nine items compared to 35 items) [43]. While our study is novel and generates a database of MS type, MS-related disability severity, and MAUI-specific estimates of relapse disutilities, there are some limitations to our research. One limitation was that the study relied on participants’ self-report of their MS-related relapse status, which may have consequences for the validity of our MS relapse status categorization scheme and the resultant disutility estimates. We had no information regarding the intensity or duration of relapse, so were unable to account for disutility impacts of these relapse features. Additionally, we could not differentiate between those people with MS experiencing a true relapse from those experiencing a “pseudo-relapse” and hence, failed to adjust our disutility estimates for the impacts of pseudo relapses. Although these are likely to be similar to MS-associated relapses as the effects on those with MS are clinically the same. The validity of our MS type categorization based on patients’ self-reports might be a minor limitation. However, the impact of this limitation is likely to be small as the measure of agreement between patient-reported and physician-reported onset phenotypes has previously been assessed in this sample at 90% and found similar to the measure of agreement (90%) between two physician reports [5]. Because our estimation of relapse disutility relied upon responses from people completing the survey while they have an ongoing relapse, people experiencing a severe relapse at the time of survey are less likely to be included in the analysis, which may have resulted in an under-estimation of the disutility of relapse.

Finally, as expected, we had a low number of people with SPMS in the ‘no’ and ‘mild’ disability categories, which inhibited the estimation of relapse disutilities for no disability group and increased the confidence intervals for effect sizes in the mild disability group. Moreover, disability severity-specific relapse disutilities for ‘unsure group’ were not entirely reliable owing to small sample limitations. In conclusion, our study provides important data on overall and MS-related disability severity-specific relapse disutilities by MS types using four MAUIs, suggesting a significant association of both the type and severity of MS with HSU decrements due to experiencing a MS-related relapse. Our estimates of relapse disutilities by disability severity provide much needed disutility weights for future multi-state health economic models of MS in Australia and other similar populations. Future comprehensive studies of relapse disutilities by MS type and disability severity, particularly those based on larger samples and clinically confirmed diagnoses of relapse status and severity, in other parts of the world are recommended to validate our baseline findings. Future work to explore the impact of inter-MAUI utility weights differences on disutilities and to investigate the preferential sensitivity of different MAUIs is also suggested. While our study’s focus was on the investigation of the disutility impacts of MS-related relapses, the evaluation of the impact of relapse on individual health dimensions of MAUIs is important and should be considered in future research to explore which aspects of HRQoL are most affected by relapses in MS population. This exercise will be helpful in identifying the physical and psychosocial health drivers of inter-MS-type HRQoL differences. Our study supports an increased and targeted support to maintain HRQoL in MS by preventing and/or optimally managing MS relapses, particularly in those living with ‘no’ and ‘severe’ disability.

### Supplementary Information

Below is the link to the electronic supplementary material.

Supplementary file1 (DOCX 46 KB)

Supplementary file2 (DOCX 20 KB)

Supplementary file3 (DOCX 15 KB)

Supplementary file4 (DOCX 17 KB)

Supplementary file5 (DOCX 17 KB)

Supplementary file6 (DOCX 23 KB)

## Data Availability

The data that support the findings of this study are not publically available, but can be made available upon reasonable request and with permission from the Australian Multiple Schelrosis Longitudinal Study (AMSLS) data custodians.
