# Supplementary material for: Estimating the disutility of relapse in relapsing–remitting and secondary progressive multiple sclerosis using the EQ-5D-5L, AQoL-8D, EQ-5D-5L-psychosocial, and SF-6D: implications for health economic evaluation models
Source: Qual Life Res. 2023 Jul 31;32(12):3373–87. doi: 10.1007/s11136-023-03486-y (PMC10624739; doi:10.1007/s11136-023-03486-y)
Supplement: Supplementary file 1 — Supplementary file1 (DOCX 46 KB) [file 11136_2023_3486_MOESM1_ESM.docx]

| **Author** | **Year** | **Country** | **Sample ^#^** | **Instruments** | **MS type**  **(all or subset) *** | **% Patients experiencing relapse(s)** | **Mean Relapse Disutility** | **Relapse Disutility by EDDS level** |
| --- | --- | --- | --- | --- | --- | --- | --- | --- |
| Grima^1^ | 2000 | Canada | 195 (42 in relapse and 153 remission) | HUI Mark 2 | RRMS (with EDSS <7) | 28.8 (prior 6 months) | NR | For patients with an EDSS 1–2: 0.1 For patients with an EDSS 3–4: 0.05 For patients with an EDSS 5–6: 0.05 (‘remission’ vs ‘relapse’ group) |
| Parkin^2^ | 2000 | UK | 50 (26 recent relapse and 24 remission) | EQ-5D | RRMS | 39.0 (prior 6 months) | 0.468 (‘remission’ vs ‘relapse’ group) | NR |
| Henriksson^3^ | 2001 | Sweden | 413 | EQ-5D | RRMS 34% PPMS 26% SPMS 37% No classification 3% | 9.0% (had a relapse last month) | 0.0635 (‘remission’ vs ‘relapse’ group) | NR |
| Prosser^4^ | 2003 | USA | 62 RRMS 67 general | Standard gamble | RRMS | NR | NR | NR |
| Kobelt^5^ | 2006 | UK | 2048 | EQ-5D | RRMS 35.5% Progressive MS 64.5% | 28.9 (prior 3 months) | 0.08 (with vs without relapses) | NR |
| Kobelt^6^ | 2006 | Switzerland | 1101 | EQ-5D | RRMS 29.1% Progressive MS 56.5% Don't know 9.6% No answer 4.8% | 16.3 (prior 3 months) | 0.08 (with vs without relapses) | NR |
| Berg^7^ | 2006 | Sweden | 1339 | EQ-5D | RRMS 21.4% Progressive MS 67.7% Don't know 9.0% No answer 1.9% | 18.0 (prior 3 months) | 0.088 (with vs without relapses) | For patients with an EDSS ≥5: 0.029 |
| Kobelt^8^ | 2006 | Austria | 1019 | EQ-5D | RRMS 35.6% Progressive MS 55.1% Don't know 7.5% No answer 1.9% | 16.8 (prior 3 months) | 0.1 (with vs without relapses) | NR |
| Kobelt^9^ | 2006 | Belgium | 799 | EQ-5D | RRMS 38.2% Progressive MS 45.7% Don’t know 13.6% No answer 2.5% | 21.5 (prior 3 months) | 0.1 (with vs without relapses) | NR |
| Kobelt^10^ | 2006 | Spain | 1 848 | EQ-5D | RRMS 37.3% Progressive MS 45.5% Don't know 12.3% No answer 5.0% | 22.6 (prior 3 months) | 0.1 (with vs without relapses) | The utility loss was similar for patients at all levels of disease severity |
| Kobelt^11^ | 2006 | Germany | 2 793 | EQ-5D | RRMS 39.7% Progressive MS 47.4% Don't know 8.8% No answer 4.0% | 24.4 (prior 3 months) | 0.1 (with vs without relapses) | For patients with an EDSS < 5: 0.09 For patients with an EDSS ≥5: 0.05 |
| Kobelt^12^ | 2006 | Netherlands | 1 549 | EQ-5D | RRMS 28.9% Progressive MS 48.6% Don't know 20.5% No answer 2.0% | 29.9 (prior 3 months) | 0.16 (with vs without relapses) | For patients with an EDSS <5: 0.16  For patients with an EDSS ≥5: 0.13 |
| Kobelt^13^ | 2006 | Italy | 921 | EQ-5D | RRMS 35.4% Progressive MS 50.7% Don't know 11.0% No answer 2.9% | 21.5 (prior 3 months) | 0.18 (with vs without relapses) | NR |
| Kobelt^14^ | 2006 | USA | 1878 | EQ-5D | PPMS 10.5 RRMS 47.6 SPMS 33.3 Not sure 8.5 | 28.8 (prior 3 months) | 0.094 (with vs without relapses) | NR |
| Orme^15^ | 2007 | UK | 2,048 | EQ-5D | RRMS 35.5 SPMS 37.2 PPMS 27.3 | 28.9 (prior 3 months) | 0.071 (with vs without relapses) | NR |
| Karampampa^16^ | 2012 | Canada | 134 | EQ-5D | RRMS | NR (prior 12 months) | 0.014  RRMS with EDSS ≤ 5 (with vs without relapses) | NR |
| Oleen-Burkey^17^ | 2012 | USA | 711 | EQ-5D | RRMS | 67.0 (prior 12 months) | 0.09 (relapse patients vs clinically stable patients) | NR |
| Hawton^18^ | 2016 | UK | 1441 | EQ-5D, SF-6D | RRMS 42.0 PPMS 19.4 SPMS 17.0 Benign 3.3 not known 18.4 | NR (prior 6 months) | 0.076 (EQ-5D) 0.052 (SF-6D) (with vs without a relapse) | NR |
| Ruutiainen^19^ | 2016 | Finland | 244 | EQ-5D-3L | RRMS | 21.5 (prior 12 month) | 0.066  RRMS with EDSS ≤ 5 (with vs without relapses) | Within each individual EDSS category up to EDSS score 5, utilities were consistently lower among those with relapse in all individual EDSS categories, except in EDSS 4 (*exact estimates were not reported*). |
| Kobelt^20^ | 2019 | Brazil | 694 | EQ-5D-3L | RRMS (74.8%) | 18.9 (prior 3 months) | 0.09  RRMS with EDSS ≤ 5 (with vs without relapses) | NR |
| Hernandez^21^ | 2021 | USA | 694 | SF-6D | RRMS 39.5 SPMS 60.5 | NR (prior 6 months) | RRMS 0.0243 SPMS 0.0405 (with vs without relapses) | NR |

**Reference**

1. Grima DT, Torrance GW, Francis G, et al. Cost and health related quality of life consequences of multiple sclerosis. Mult Scler. 2000; 6: 91-8.

2. Parkin D, Jacoby A, McNamee P, et al. Treatment of multiple sclerosis with interferon beta: an appraisal of cost-effectiveness and quality of life. J Neurol Neurosurg Psychiatry. 2000; 68: 144-9.

3. Henriksson F, Fredrikson S, Masterman T, et al. Costs, quality of life and disease severity in multiple sclerosis: a cross-sectional study in Sweden. Eur J Neurol. 2001; 8: 27-35.

4. Prosser LA, Kuntz KM, Bar-Or A, et al. Patient and community preferences for treatments and health states in multiple sclerosis. Mult Scler. 2003; 9: 311-9.

5. Kobelt G, Berg J, Lindgren P, et al. Costs and quality of life of multiple sclerosis in the United Kingdom. Eur J Health Econ. 2006; 7 Suppl 2: S96-104.

6. Kobelt G, Berg J, Lindgren P, et al. Costs and quality of life of multiple sclerosis in Switzerland. Eur J Health Econ. 2006; 7 Suppl 2: S86-95.

7. Berg J, Lindgren P, Fredrikson S, et al. Costs and quality of life of multiple sclerosis in Sweden. Eur J Health Econ. 2006; 7 Suppl 2: S75-85.

8. Kobelt G, Berg J, Lindgren P, et al. Costs and quality of life of multiple sclerosis in Austria. Eur J Health Econ. 2006; 7 Suppl 2: S14-23.

9. Kobelt G. Costs and quality of life for patients with multiple sclerosis in Belgium. Eur J Health Econ. 2006; 7 Suppl 2: S24-33.

10. Kobelt G, Berg J, Lindgren P, et al. Costs and quality of life of multiple sclerosis in Spain. Eur J Health Econ. 2006; 7 Suppl 2: S65-74.

11. Kobelt G, Berg J, Lindgren P, et al. Costs and quality of life of multiple sclerosis in Germany. Eur J Health Econ. 2006; 7 Suppl 2: S34-44.

12. Kobelt G, Berg J, Lindgren P, et al. Costs and quality of life in multiple sclerosis in The Netherlands. Eur J Health Econ. 2006; 7 Suppl 2: S55-64.

13. Kobelt G, Berg J, Lindgren P, et al. Costs and quality of life of multiple sclerosis in Italy. Eur J Health Econ. 2006; 7 Suppl 2: S45-54.

14. Kobelt G, Berg J, Atherly D, et al. Costs and quality of life in multiple sclerosis: a cross-sectional study in the United States. Neurology. 2006; 66: 1696-702.

15. Orme M, Kerrigan J, Tyas D, et al. The effect of disease, functional status, and relapses on the utility of people with multiple sclerosis in the UK. Value Health. 2007; 10: 54-60.

16. Karampampa K, Gustavsson A, Miltenburger C, et al. Treatment experience, burden, and unmet needs (TRIBUNE) in multiple sclerosis: the costs and utilities of MS patients in Canada. J Popul Ther Clin Pharmacol. 2012; 19: e11-25.

17. Oleen-Burkey M, Castelli-Haley J, Lage MJ, et al. Burden of a multiple sclerosis relapse: the patient's perspective. Patient. 2012; 5: 57-69.

18. Hawton A, Green C. Health Utilities for Multiple Sclerosis. Value Health. 2016; 19: 460-8.

19. Ruutiainen J, Viita AM, Hahl J, et al. Burden of illness in multiple sclerosis (DEFENSE) study: the costs and quality-of-life of Finnish patients with multiple sclerosis. J Med Econ. 2016; 19: 21-33.

20. Kobelt G, Teich V, Cavalcanti M, et al. Burden and cost of multiple sclerosis in Brazil. PLoS One. 2019; 14: e0208837.

21. Hernandez L, O'Donnell M, Postma M. Predictors of Health Utility in Relapsing-Remitting and Secondary-Progressive Multiple Sclerosis: Implications for Future Economic Models of Disease-Modifying Therapies. Pharmacoeconomics. 2021; 39: 243-56.
