# Supplementary material for: Estimating the disutility of relapse in relapsing–remitting and secondary progressive multiple sclerosis using the EQ-5D-5L, AQoL-8D, EQ-5D-5L-psychosocial, and SF-6D: implications for health economic evaluation models
Source: Qual Life Res. 2023 Jul 31;32(12):3373–87. doi: 10.1007/s11136-023-03486-y (PMC10624739; doi:10.1007/s11136-023-03486-y)
Supplement: Supplementary file 2 — Supplementary file2 (DOCX 20 KB) [file 11136_2023_3486_MOESM2_ESM.docx]

| **Supplement 2.** Comparisons of the dimensions and content of the four multi-attribute utility instruments used in this study. | | | | |
| --- | --- | --- | --- | --- |
|  | EQ-5D-5L | AQoL-8D | SF-6D | EQ-5D-5L-Psychosocial |
|  |  |  |  |  |
| Year (preference weights available) | 2012 | 2009 | 2002 | 2020 |
| Countries of origin | Europe/UK | Australia | UK/USA | Europe/UK/Australia |
| Dimensions | 5 | 8 | 6 | 9 |
| Items | 5 | 35 (25 related to psychosocial health) | 6 | 9 |
| Response levels | 5 | 4-6 | 4-6 | 4-6 |
| States defined | 3,125 | 2.4 x 10^23^ | 18,000 | 1,953,125 |
| Total time taken | 1 minute | 5.5 minutes | 2.5 minutes | 1-2 minutes |
| **Physical health-related items** |  |  |  |  |
| *Physical ability/mobility/vitality/coping/control* | 1 | 3 | 1 | 1 |
| *Bodily function/self-care* | 1 | 1 |  | 1 |
| *Pain/discomfort* | 1 | 2 | 1 | 1 |
| *Senses* |  | 2 |  | 1 |
| *Usual activities/work* | 1 | 4 | 1 | 1 |
| *Communication* |  | 1 |  |  |
| **Psychosocial health-related items** |  |  |  |  |
| *Sleeping* |  | 1 |  | 1 |
| *Depression/anxiety/anger* | 1 | 7 | 1 |  |
| *General satisfaction* |  | 4 |  |  |
| *Self-esteem* |  | 2 |  |  |
| *Cognition/memory ability* |  |  |  |  |
| *Social function/relationships* |  | 6 | 1 | 1 |
| *(Family) role* |  | 1 | 1 |  |
| *Intimacy/sexual relationships* |  | 1 |  |  |
| *Vitality* |  | 1 |  | 1 |
| *Community Connectedness* |  | 1 |  | 1 |
| Abbreviation: AQoL-8D, Assessment of Quality of Life-8 dimensions; SF-6D, Short Form 6-dimension.  Sources: (1) *Richardson (2011).* *Review and Critique of Health Related Multi Attribute Utility Instruments. Centre for Health Economics. Research paper 2011(64).* (2) *Richardson (2015). Why do multi-attribute utility instruments produce different utilities: the relative importance of the descriptive systems, scale and 'micro-utility' effects. Qual Life Res. 2015 Aug;24(8):2045-53*. (3) *Chen G, Olsen JA. Filling the psycho-social gap in the EQ-5D: the empirical support for four bolt-on dimensions. Quality of Life Research. 2020 Nov;29(11):3119-29.* | | | | |
