# Supplementary material for: Estimating the disutility of relapse in relapsing–remitting and secondary progressive multiple sclerosis using the EQ-5D-5L, AQoL-8D, EQ-5D-5L-psychosocial, and SF-6D: implications for health economic evaluation models
Source: Qual Life Res. 2023 Jul 31;32(12):3373–87. doi: 10.1007/s11136-023-03486-y (PMC10624739; doi:10.1007/s11136-023-03486-y)
Supplement: Supplementary file 3 — Supplementary file3 (DOCX 15 KB) [file 11136_2023_3486_MOESM3_ESM.docx]

Supplement 3. The Approximate EDSS equivalents of the Patient Determined Disease Steps (PDDS)

| **Patient Determined Disease Steps (Description)** | **Approximate EDSS Equivalent** | **Broad Disability Category** | **Sample Size per group*** |
| --- | --- | --- | --- |
| **0** (I may have some mild symptoms, mostly sensory, due to MS but they do not limit my activity or lifestyle) | 0 | No Disability | 346 |
| **1** (I have some noticeable symptoms from my MS, but they are minor and have only a small effect on my lifestyle) | 1 | Mild Disability | 293 |
| **2** (I don't have any limitations in my walking ability. However, I do have problems due to MS that limit daily activities in other ways) | 2-3 |  |  |
| **3** (MS does interfere with my activities, especially walking. I can work a full day, but athletic or physically demanding activities are more difficult than they used to be. I usually don’t need to use a walking stick (cane) or other walking aid, but I might during an MS attack) | 4-5 | Moderate Disability | 462 |
| **4** (I can walk about 8 meters (or 25 feet) without using a walking stick (cane) or other walking aid such as a splint, brace, or crutch, but I may use a walking aid for greater distances) | 6 |  |  |
| **5** (To be able to walk 8 meters (or 25 feet), I have to have a walking stick (cane), a single crutch, or someone to hold onto. I can get around the house by holding onto furniture or touching the walls for support. I may use a scooter or wheelchair for greater distances) | 6 |  |  |
| **6** (To walk 8 meters (or 25 feet), I must have two walking sticks (canes), two crutches, or a walking frame (walker). I may use a scooter or wheelchair for greater distances) | 6.5 | Severe Disability | 184 |
| **7** (My main form of mobility is a wheelchair. I may be able to stand and/or take one or two steps, but I can’t walk 8 meters (or 25 feet), even with crutches or a walking frame) | 7 |  |  |
| **8** (I am unable to sit in a wheelchair for more than 1 hour, and I spend most of my time in bed) | 8-9 |  |  |
|  | | | |
