# Supplementary material for: Estimating the disutility of relapse in relapsing–remitting and secondary progressive multiple sclerosis using the EQ-5D-5L, AQoL-8D, EQ-5D-5L-psychosocial, and SF-6D: implications for health economic evaluation models
Source: Qual Life Res. 2023 Jul 31;32(12):3373–87. doi: 10.1007/s11136-023-03486-y (PMC10624739; doi:10.1007/s11136-023-03486-y)
Supplement: Supplementary file 4 — Supplementary file4 (DOCX 17 KB) [file 11136_2023_3486_MOESM4_ESM.docx]

**Supplement 4:** Clinical and sociodemographic characteristics of the QoL Survey 2020 respondents versus non-respondents

| **N=2,513** | Respondents | Non-respondents |
| --- | --- | --- |
| **Characteristics** | (N=1683) | (N=830) |
| **Age at the time of survey** |  |  |
| Average in years (n) | 58.6 (1683) | 55.6 (830) |
| **Sex** |  |  |
| Male % (n) | 20.4 (343) | 21.8 (181) |
| Female % (n) | 79.6 (1340) | 78.2 (649) |
| **Age group** |  |  |
| <35 % (n) | 1.8 (31) | 4.7 (39) |
| 35-44 % (n) | 9.3 (157) | 15.5 (129) |
| 45-54 % (n) | 24.7 (415) | 23.5 (195) |
| 55-64 % (n) | 32.1 (540) | 30.8 (256) |
| 65+ % (n) | 32.1 (540) | 25.4 (211) |
| **State/Territory of usual residence** |  |  |
| New South Wales % (n) | 28.1 (473) | 32.2 (267) |
| Victoria % (n) | 29.3 (493) | 23.6 (196) |
| Queensland % (n) | 12.4 (209) | 18.6 (154) |
| South Australia % (n) | 9.9 (167) | 8.4 (70) |
| Western Australia % (n) | 9.8 (165) | 9.4 (78) |
| Australian Capital Territory % (n) | 4.0 (67) | 3.0 (25) |
| Tasmania % (n) | 5.7 (96) | 4.2 (35) |
| Northern Territory % (n) | <1.0 (1) | <1.0 (3) |
| **Education Level** |  |  |
| Primary % (n) | <1.0 (8) | <1.0 (7) |
| Secondary % (n) | 24.6 (400) | 26.1 (203) |
| Occupation Certificate % (n) | 33.5 (545) | 30.3 (236) |
| University (bachelors) % (n) | 21.0 (341) | 22.3 (174) |
| University (Postgrad) % (n) | 16.7 (272) | 16.9 (132) |
| Other % (n) | 3.7 (60) | 3.5 (27) |
| **MS type** |  |  |
| PPMS % (n) | 11.3 (190) | NA |
| RRMS % (n) | 62.8 (1,056) | NA |
| SPMS % (n) | 14.2 (239) | NA |
| PRMS % (n) | 2.5 (42) | NA |
| Unsure % (n) | 8.0 (134) | NA |
| **Disability severity** |  |  |
| No disability % (n) | 23.9 (402) | NA |
| Mild disability % (n) | 20.4 (343) | NA |
| Moderate disability % (n) | 36.1 (608) | NA |
| Severe disability % (n) | 18.8 (317) | NA |
| **MS duration since diagnosis** |  |  |
| Average in years (n) | 19.0 (1393) | 16.9 (683) |
| *Notes:* NA=not available; MS= multiple sclerosis | | |
|  | | |
