# Supplementary material for: Estimating the disutility of relapse in relapsing–remitting and secondary progressive multiple sclerosis using the EQ-5D-5L, AQoL-8D, EQ-5D-5L-psychosocial, and SF-6D: implications for health economic evaluation models
Source: Qual Life Res. 2023 Jul 31;32(12):3373–87. doi: 10.1007/s11136-023-03486-y (PMC10624739; doi:10.1007/s11136-023-03486-y)
Supplement: Supplementary file 5 — Supplementary file5 (DOCX 17 KB) [file 11136_2023_3486_MOESM5_ESM.docx]

**Supplement 5**. Unadjusted mean (95% confidence interval) disutilities of current relapse in people with RRMS, SPMS and ROMS

|  | RRMS (n=1,056) | | | |  | SPMS (n=239) | |  | ROMS (N=1,295) | |
| --- | --- | --- | --- | --- | --- | --- | --- | --- | --- | --- |
|  | Mean (95%CI) | | *P* value | |  | Mean (95%CI) | *P* value |  | Mean (95%CI) | *P* value |
| **EQ-5D-5L** |  | |  | |  |  |  |  |  |  |
| No | (Ref.) | | | | | | | | | |
| Yes | **-0.196 (-0.241, -0.152)** | | **<0.001** | |  | **-0.150 (-0.257, -0.043)** | **0.006** |  | **-0.222 (-0.268, -0.176)** | **<0.001** |
| Unsure | **-0.205 (-0.259, -0.151)** | | **<0.001** | |  | **-0.154 (-0.273, -0.036)** | **0.011** |  | **-0.241 (-0.296, -0.186)** | **<0.001** |
| **AQoL-8D** |  | |  | |  |  |  |  |  |  |
| No | (Ref.) | | | | | | | | | |
| Yes | **-0.175 (-0.211, -0.138)** | | **<0.001** | |  | **-0.167 (-0.228, -0.105)** | **<0.001** |  | **-0.186 (-0.218, -0.154)** | **<0.001** |
| Unsure | **-0.140 (-0.184, -0.096)** | | **<0.001** | |  | **-0.098 (-0.167, -0.031)** | **0.005** |  | **-0.146 (-0.184, -0.108)** | **<0.001** |
| **EQ-5D-5L-Psychosocial** | | | |  |  |  |  |  |  |  |
| No | | (Ref.) | | | | | | | | |
| Yes | | **-0.152 (-0.186, -0.119)** | | **<0.001** |  | **-0.138 (-0.198, -0.078)** | **<0.001** |  | **-0.163 (-0.193, -0.133)** | **<0.001** |
| Unsure | | **-0.145 (-0.185, -0.105)** | | **<0.001** |  | **-0.095 (-0.162, -0.029)** | **0.005** |  | **-0.149 (-0.184, -0.114)** | **<0.001** |
| **SF-6D** |  | |  | |  |  |  |  |  |  |
| No | (Ref.) | | | | | | | | | |
| Yes | **-0.231 (-0.280, -0.182)** | | **<0.001** | |  | **-0.164 (-0.251, -0.078)** | **<0.001** |  | **-0.236 (-0.280, -0.192)** | **<0.001** |
| Unsure | **-0.199 (-0.259, -0.140)** | | **<0.001** | |  | -0.084 (-0.180, 0.012) | 0.086 |  | **-0.195 (-0.247, -0.143)** | **<0.001** |
| *Abbreviations*: MS = multiple sclerosis; RRMS = relapsing remitting MS; SPMS = Secondary Progressive MS; ROMS = Relapse Onset MS (which includes both RRMS and SPMS) | | | | | | | | | | |
