# Supplementary material for: Estimating the disutility of relapse in relapsing–remitting and secondary progressive multiple sclerosis using the EQ-5D-5L, AQoL-8D, EQ-5D-5L-psychosocial, and SF-6D: implications for health economic evaluation models
Source: Qual Life Res. 2023 Jul 31;32(12):3373–87. doi: 10.1007/s11136-023-03486-y (PMC10624739; doi:10.1007/s11136-023-03486-y)
Supplement: Supplementary file 6 — Supplementary file6 (DOCX 23 KB) [file 11136_2023_3486_MOESM6_ESM.docx]

**Supplement 6.** unadjusted mean (95% confidence intervals) disutilities of relapse in MS subgroups, by disability severity

|  |  | **No Disability** | **Mild Disability** | **Moderate Disability** | **Severe Disability** |
| --- | --- | --- | --- | --- | --- |
| **RRMS** | **EQ-5D-5L** | | | | |
|  | No | Ref. | Ref. | Ref. | Ref. |
|  | Yes | **-0.166 (-0.257, -0.076)** | -0.053 (-0.122, 0.016) | **-0.114 (-0.167, -0.061)** | -0.062 (-0.283, 0.159) |
|  | Unsure | **-0.159 (-0.242, -0.076)** | **-0.129 (-0.227, -0.031)** | **-0.070 (-0.136, -0.005)** | -0.175 (-0.408, 0.565) |
|  | **AQoL-8D** | | | | |
|  | No | Ref. | Ref. | Ref. | Ref. |
|  | Yes | **-0.127 (-0.221, -0.033)** | -0.048 (-0.111, 0.016) | **-0.099 (-0.145, -0.054)** | **-0.126 (-0.221, -0.033)** |
|  | Unsure | **-0.148 (-0.233, -0.062)** | -0.055 (-0.146, 0.037) | -0.027 (-0.083, 0.029) | **-0.157 (-0.293, -0.021)** |
|  | **EQ-5D-5L-Psychosocial** | | | | |
|  | No | (Ref.) | | | |
|  | Yes | **-0.107 (-0.194, -0.020)** | -0.043 (-0.102, 0.015) | **-0.078 (-0.120, -0.035)** | -0.117 (-0.236, 0.002) |
|  | Unsure | **-0.150 (-0.230, -0.071)** | -0.098 (-0.181, 0.014) | -0.038 (-0.089, 0.013) | -0.105 (-0.230, 0.019) |
|  | **SF-6D** | | | | |
|  | No | Ref. | Ref. | Ref. | Ref. |
|  | Yes | **-0.241 (-0.360, -0.123)** | -0.067 (-0.156, 0.021) | **-0.080 (-0.139, -0.021)** | **-0.262 (-0.418, -0.105)** |
|  | Unsure | **-0.217 (-0.326, -0.109)** | **-0.159 (-0.283, -0.034)** | -0.005 (-0.077, 0.066) | **-0.181 (-0.345, -0.017)** |
|  |  |  |  |  |  |
| **SPMS** | **EQ-5D-5L** | | | | |
|  | No | Ref. | Ref. | Ref. | Ref. |
|  | Yes | 0(0) ^ | -0.219 (-0.714, 0.277) | -0.092 (-0.204, 0.020) | **-0.275 (-0.413, -0.137)** |
|  | Unsure | 0(0) ^ | -0.439 (-1.011, 0.134) | -0.025 (-0.156, 0.105) | **-0.248 (-0.392, -0.104)** |
|  | **AQoL-8D** | | | | |
|  | No | Ref. | Ref. | Ref. | Ref. |
|  | Yes | 0(0) ^ | -0.068 (-0.371, 0.235) | **-0.153 (-0.239, -0.067)** | **-0.195 (-0.286, -0.103)** |
|  | Unsure | 0(0) ^ | -0.197 (-0.547, 0.152) | -0.032 (-0.135, 0.070) | **-0.137 (-0.230, -0.043)** |
|  | **EQ-5D-5L-Psychosocial** | | | | |
|  | No | (Ref.) | | | |
|  | Yes | 0(0) ^ | -0.083 (-0.403, 0.237) | **-0.098 (-0.180, -0.016)** | **-0.188 (-0.275, -0.101)** |
|  | Unsure | 0(0) ^ | -0.179 (-0.549, 0.191) | -0.006 (-0.102, 0.090) | **-0.156 (-0.246, -0.065)** |
|  | **SF-6D** |  |  |  |  |
|  | No | Ref. | Ref. | Ref. | Ref. |
|  | Yes | 0(0) ^ | -0.046 (-0.412, 0.320) | -0.104 (-0.211, -0.002) | **-0.264 (-0.401, -0.127)** |
|  | Unsure | 0(0) ^ | 0.137 (-0.422, 0.697) | -0.109 (-0.236, 0.017) | -0.0619 (-0.199, 0.076) |
|  |  |  |  |  |  |
| **RRMS + SPMS** | **EQ-5D-5L** |  |  |  |  |
|  | No | Ref. | Ref. | Ref. | Ref. |
|  | Yes | **-0.166 (-0.257, -0.076)** | -0.064 (-0.133, 0.005) | **-0.110 (-0.159, -0.062)** | **-0.211 (-0.328, -0.093)** |
|  | Unsure | **-0.159 (-0.242, -0.076)** | **-0.161 (-0.257, -0.065)** | **-0.064 (-0.123, -0.004)** | **-0.228 (-0.351, -0.104)** |
|  | **AQoL-8D** |  |  |  |  |
|  | No | Ref. | Ref. | Ref. | Ref. |
|  | Yes | **-0.127 (-0.221, -0.033)** | -0.047 (-0.108, 0.014) | **-0.111 (-0.152, -0.071)** | **-0.173 (-0.247, -0.099)** |
|  | Unsure | **-0.148 (-0.233, -0.062)** | -0.069 (-0.154, 0.017) | -0.029 (-0.078, 0.021) | **-0.142 (-0.219, -0.066)** |
|  | **EQ-5D-5L-Psychosocial** | | | | |
|  | No | (Ref.) | | | |
|  | Yes | **-0.107 (-0.194, -0.020)** | -0.044 (-0.100, 0.012) | **-0.083 (-0.120, -0.045)** | **-0.166 (-0.236, -0.096)** |
|  | Unsure | **-0.150 (-0.230, -0.071)** | **-0.103 (-0.182, -0.025)** | -0.032 (-0.077, 0.013) | **-0.140 (-0.213, -0.067)** |
|  | **SF-6D** |  |  |  |  |
|  | No | Ref. | Ref. | Ref. | Ref. |
|  | Yes | **-0.241 (-0.360, -0.123)** | -0.066 (-0.151, 0.019) | **-0.086 (-0.137, -0.034)** | **-0.263 (-0.368, -0.159)** |
|  | Unsure | **-0.217 (-0.326, -0.109)** | **-0.139 (-0.259, -0.019)** | -0.029 (-0.092, 0.033) | -0.205 (-0.345, 0.007) |
| ^n=0 participants had relapse in this category, No disability includes [Expanded Disability Status Scale](https://www.sciencedirect.com/topics/medicine-and-dentistry/expanded-disability-status-scale) (EDSS) level 0, Mild includes EDSS levels 1–3.5, moderate includes levels 4–6 and severe includes levels 6.5–9.5.  *Abbreviations*: MS=multiple sclerosis; RRMS=relapsing remitting MS; SPMS=secondary progressive MS; ROMS=relapse onset MS. | | | | | |
